# News and Coming Events

**Published:** 1908-02-22

**Authors:** 


### NEWS AND COMING EVENTS.

Dr. W. P. S. Branson has been appointed physician to the Royal Free Hospital, Gray's Inn Road.

Dr. Bernard H. Wedd has been appointed Bacteriologist to the Royal London Ophthalmic Hospital, E.C.

Dr. R. C. Jewesbury has been appointed Physician with charge of out-patients at the Victoria Hospital for Children, Chelsea.

The fourth international congress on radiography and electro therapeutics will be held at Amsterdam on September 1 to 5 next.

The thirty-seventh congress of the German Surgical Society will take place at Berlin in the third week in April, commencing April 21 next.

A public meeting in support of the State registration of trained nurses was held at the Caxton Hall, Westminster, on Friday afternoon, February 21. Lady Helen Munro Ferguson presided.

The Council of the Royal College of Surgeons of England has appointed Mr. Arthur Keith, F.R.C.S., to the office of Conservator of the Museum, rendered vacant by the death of Professor Charles Stewart.

Last week's death-rate in London showed a sharp rise from 17.7 to 19.0 per thousand. Eighty-four deaths in the Metropolis were attributed directly to influenza, against 25, 32, and 34 in the preceding three weeks.

The managers of the Edinburgh Royal Infirmary have placed Mr. Dowden in charge of the late Professor Annandale's wards, and he will act for the time being as professor of surgery until the vacancy has been definitely filled by the appointment of Professor Annandale's successor.

Lord Cawdor, the Treasurer of the London Homœopathic Hospital, Great Ormond Street, W.C., has received the sum of £1,000 for the naming of the first female bed in the new extension, to be called the William and Charlotte Clauson-Thue Bed, in memory of the late Mr. and Mrs. Clauson-Thue.

At the present time the first M.B. examination of the University of Cambridge is held twice in the year, in June and December. It is now proposed to hold a third examination at the beginning of the Michaelmas term, and it is suggested that the examination shall begin on the Tuesday or Friday (which shall first happen) after October 2.

At the annual general meeting of the Harveian Society the following officers were elected for the ensuing year : President, Mr. D'Arcy Power; Vice-Presidents, Dr. G. A. Sutherland, Dr. C. Buttar, Dr. H. J. Macevoy, and Dr. W. H. Blenkinsop; Treasurer, Mr. Francis Jaffrey; Hon. Secretaries, Mr. T. Crisp English and Dr. W. H. Willcox.

At the University of Cambridge the third examination for medical and surgical degrees—Part II—(1) Surgery, (2) Midwifery, (3) Medicine—will be held on April 28, 29, and 30. The oral, practical, and clinical examinations will be held on May 1, 2, 4, 5, 6, and 7. The names of candidates should be sent to the Registrary on or before Tuesday, April 14. Their certificates, accompanied by their postal addresses, must be sent to the Registrary on or before Thursday, April 23.

The Council of the University of Sheffield has appointed Mr. John L. Annan, M.B., to the post of Demonstrator in Anatomy. The Council has also appointed the following to the newly-instituted clinical tutorships : Mr. Miles H. Phillips, M.B., F.R.C.S., in Obstetrics and Gynæcology; Mr. Graham Simpson, F.R.C.S., in Surgery; Mr. A. E. Barnes, M.B., M.R.C.P., in Medicine.

A Conversazione in connection with the therapeutical and pharmacological section of the Royal Society of Medicine will be held at the Apothecaries' Hall, Blackfriars, E.C., on Tuesday, February 25, at 4.30 p.m. Ladies are invited. There will be an address by Dr. Harry Campbell on "The Therapeutics of Diet." Demonstrations of electrical apparatus and of colour photography, and an exhibition of instruments, drugs, and foods. Admission by signature or card of Fellow of the Society or a member of Section.

The sum of £1,000, which has been collected among the patients and friends of the late Dr. W. S. Playfair, will be presented to King's College Hospital for the erection of a memorial to him at that institution. In view of the eminent services rendered to the hospital by Dr. Playfair, and in deference to the wishes of his family, the Removal Fund Committee have decided to devote this money to the building and equipment of an appropriate department of the new hospital shortly to be erected at Denmark Hill. This department will be named after him, and will bear an inscription recording his 35 years' work at King's College Hospital.

The annual general meeting of the London Fever Hospital, Islington, was held on February 14, Lord Balfour, the President, being in the chair. Sir Shirley Murphy, in moving the adoption of the report, said that, although the work of reconstructing the hospital buildings had been continued during the year, 852 patients had been under treatment, which was a larger number than in any year since 1901. Scarlet fever had been characterised in recent years in London by a low rate of mortality, but, whatever the mortality from the disease in London as a whole, the mortality at their institution had always been somewhat lower.

At the meeting of the Council of the Royal College of Surgeons of England, held on February 13, Mr. W. Watson Cheyne, C.B., F.R.S., was appointed Bradshaw Lecturer for the ensuing collegiate year, and Mr. J. Ward Cousins was re-elected the representative of the College upon the Central Midwives Board for a further period of one year. A small committee was appointed with the object of keeping the Council informed as to what is being done from time to time regarding the medical inspection of school children and the arrangements for teaching elementary hygiene in training colleges and schools.

The annual meeting of the Governors of the Royal Maternity Charity of London was held on February 11, Mr. Cornelius Barham presiding. The Secretary, Major G. L. B. Killick, read the report of the Medical Committee for 1907. Of the 2,376 cases attended by 33 midwives, the percentages of deaths per 1,000 were—mothers, 1.26, and infants, 15.74. The Medical Committee were of opinion that the deaths were all due to non-preventable causes, and that no blame was attachable to the staff of the charity. The rate of mortality among mothers and infants was exceptionally low, and spoke highly for the skill and care of the midwives employed.

The annual general meeting of Governors of the Royal London Ophthalmic Hospital (Moorfields Eye Hospital) will be held on Tuesday, February 25, at three o'clock, at the Mansion House, by kind permission of the Right Hon. the Lord Mayor, who has consented to preside.

The third annual Court of Governors of the Royal National Orthopædic Hospital will be held in the Board-room at Charing Cross Hospital, Agar Street, Strand, W.C., on Wednesday, February 26, at 4 p.m. The in-patients of the hospital are temporarily accommodated in wards which have been rented at Charing Cross Hospital, during the building of the new Orthopædic Hospital in Great Portland Street.

The little seaport town of Workington, which has lately been visited by a severe outbreak of scarlet fever, has now been thrown into a panic by an epidemic of typhoid. No fewer than fifty fresh cases have occurred during the past week. Fortunately, so far only a few have terminated fatally. To make matters worse the authorities of the local isolation hospital have had to send home all the scarlet-fever patients in order to make room for the typhoid cases—a step which it is feared will spread infection far and wide.

The Local Government Board have issued regulations as to ships arriving from foreign ports, prescribing the procedure to be followed for preventing the transmission of plague by rats on board ship. To obviate this rats are in all cases to be destroyed when the ship is infected with plague or when rats in the ship are infected with that disease. Expensive apparatus will be involved in efficiently carrying out the regulations. Nearly half a million rats have been destroyed in ships and warehouses in the Port of London since the work of extermination was begun.

Lord Rothschild, as President of the Royal Buckinghamshire Hospital, on February 14 laid the foundation-stone of the new wing of the building at Aylesbury, which is being erected to provide an operating theatre fitted up on modern lines, and also additional accommodation for the nurses. The total cost of the work, including the furnishing, will be about £10,000, towards which a sum of £7,500 has been subscribed. Among the subscriptions to this fund have been several by members of the Rothschild family, Lord Rothschild recently making a second gift of £1,000. Mr. George Carrington, the Chairman of the monthly Board, handed to Lord Rothschild a suitably inscribed silver trowel, with which he performed the ceremony of laying the foundation-stone.

Sir Richard B. Martin, Bart., Treasurer, presided, on February 13 at the annual general meeting of St. Mark's Hospital for Fistula. He pointed out that from the nature of the diseases attended to by the hospital it was necessary to keep each patient under treatment for a longer period than would be possible in a general hospital. During the past year the total number of in-patients was 563, an increase of 82 as compared with 1906. The average daily number of fully-occupied beds was 41.2, and 5,985 attendances had been made by 1,641 out-patients. The Chairman referred to the payments made by patients, and said he was strongly of opinion that all patients who could possibly pay anything should do so. The accounts show that during the year out-patients had contributed £27 5s. 6d., and in-patients £424 19s. Mr. F. Swinford Edwards, F.R.C.S. (Hon. Surgeon), said that the past year had been a very successful one from a surgical point of view.

Mr. Alfred E. Johnson, M.B., F.R.C.S., has been appointed resident medical officer to the Middlesex Hospital.

The Royal Dental Hospital, Leicester Square, has received a donation of £100, in this its jubilee year, from the Worshipful Company of Goldsmiths.

In connection with the recent outbreak of cholera among the Mecca pilgrims, further cases are reported from Hedieh, and the infected locality has in consequence been isolated by a military cordon. Quarantine stations have been established, and the railway authorities have received orders to exercise the strictest supervision over pilgrims returning from the Holy places. Observation posts have also been established at various points on the desert routes and at the bridge over the Jordan near Jericho.

In 1907 the question of the use of stimulants in hospitals was discussed in the English Press. Statistical information as to the decreased consumption was introduced. In the South Infirmary, Cork, the staff have kept pace with this development, the expenditure on stimulants having decreased, though the number of intern patients have increased, as the following figures show: In 1867 £162 17s. was spent on stimulants, while the number of intern patients was 929; in 1887 the figures were £88 17s. 4d. and 1,020; while in 1907 they were £28 5s. 8d. and 1,210.

The death of Mr. Charles Arthur Aikin, F.R.C.S., on February 11 removes one of the oldest members of the medical profession. The only son of Charles R. Aikin, M.R.C.S., he was born in London in 1821. He was educated at University College School and Guy's Hospital, and became qualified on the eve of his twenty-first birthday. Marrying young, he settled in the district known as Tyburnia, and soon formed a large practice on the north side of the park. In 1858 the Royal College of Surgeons honoured him with its Fellowship. Mr. Aikin retired from practice at the age of seventy, and spent the last years of his life in North Wales.

On Sunday last Dr. Hall-Edwards, of Birmingham, had his left hand amputated owing to the effects of X-ray dermatitis. He is reported to be making fairly good progress. Dr. Hall-Edwards was the pioneer operator with the Röntgen rays in England, and he had been engaged upon the application of electricity to surgery for several years before the announcement of Röntgen's discovery. The first radiographs for the purpose of an operation in this country were taken by him. Dr. Hall-Edwards was senior radiographer with the Imperial Yeomanry in the South African war, and he is surgeon-radiographer to the Birmingham General Hospital. His right hand is also affected, but it is hoped that this will be saved.

At the meeting of the London County Council on February 18 the receipt was announced of a letter from Dr. Henry Maudsley, of Queen Street, Mayfair, offering a contribution of £30,000 towards a scheme for the establishment in London of a specially equipped hospital for mental diseases. In his letter Dr. Maudsley stated that he believes that the establishment of such a hospital, in close touch with the general hospitals and the University of London, would do much to break down the unfortunate isolation from general medical knowledge and research in which the study and treatment of insanity remains. A vote of thanks was passed to Dr. Maudsley for his generous offer, and the matter was referred to the Asylums Committee.

